# Quantitative, Spatially Defined Expression of Leukocyte-associated Immunoglobulin-like Receptor in Non–small Cell Lung Cancer

**DOI:** 10.1158/2767-9764.CRC-22-0334

**Published:** 2023-03-21

**Authors:** Thazin N. Aung, Niki Gavrielatou, Ioannis A. Vathiotis, Aileen I. Fernandez, Saba Shafi, Vesal Yaghoobi, Sneha Burela, Tyler MacNeil, Fahad Shabbir Ahmed, Han Myint, Dallas B. Flies, Solomon Langermann, David L. Rimm

**Affiliations:** 1 Department of Pathology, Yale University School of Medicine, New Haven, Connecticut.; 2 Department of Medicine, School of Medicine, National and Kapodistrian University of Athens, Athens, Greece.; 3 NextCure Inc, Beltsville, Maryland.; 4 Department of Medicine, Yale University School of Medicine, New Haven, Connecticut.

## Abstract

Targeting the interaction of leukocyte-associated immunoglobulin-like receptor-1 (LAIR-1) and its ligands has been shown to reinstate antitumor immunity. In addition, the introduction of the LAIR-1 decoy protein, LAIR-2, sensitizes previously resistant lung tumors to programmed death-1 (PD-1) blockade, indicating the potential of LAIR-1 as an alternative marker for anti-PD-1 resistance in lung cancer. Here, we assessed LAIR-1 as compared with programmed death-ligand 1 (PD-L1) expression in various tumors, with a focus on non–small cell lung cancer (NSCLC) and its histologic subtypes using multiplexed quantitative immunofluorescence (mQIF) in 287 (discovery cohort) and 144 (validation cohort) patients with NSCLC. In addition, using multispectral imaging technology on mQIF images, we evaluated the localization of LAIR-1 on various cell types. We observed that CD14^+^, CD68^+^, and CD163^+^ monocytes and CK^+^ tumor cells predominantly expressed LAIR-1 more than other cell types. Furthermore, LAIR-1 expression in the tumor compartment was significantly higher in patients with lung adenocarcinoma (LUAD) than those with lung squamous cell carcinoma subtype (**, *P* = 0.003). Our results indicated that high tumor LAIR-1 expression in patients with LUAD is negatively associated with OS (overall survival, HR = 2.4; *, *P* = 0.02) highlighting its prognostic value in LUAD but not in other subtypes. The Pearson correlation between LAIR-1 and PD-L1 is 0.31; however, mutual exclusive staining pattern (i.e., several cases were positive for LAIR-1 and negative for PD-L1) was observed. Altogether, our data suggest that the combination therapy of anti-PD-1/PD-L1 with anti-LAIR-1 or the anti-LAIR-1 monotherapy alone may be promising cancer immunotherapeutic strategies.

**Significance:**

The spatial, quantitative assessment of LAIR-1 in NSCLC shows positive association of OS with high LAIR-1^+^/CD68^+^ cell densities and negative association of OS with high LAIR-1 expression in LUAD tumor subtype.

## Introduction

LAIR-1 also known as CD305, is a 32 kDa transmembrane glycoprotein with an immunoglobulin-like domain and a cytoplasmic tail containing two immunoreceptor tyrosine based motifs (ITIM; ref. 1). It is encoded by *LAIR-1* gene which maps to a region of 19q13.4 (leukocyte receptor cluster), a member of both the immunoglobulin superfamily and the leukocyte-associated inhibitory receptor family. Its expression has been reported in many the immune cell subsets and thought to be restricted to immune cells (1, 2). Collagen-induced CD8^+^ T-cell exhaustion in cancer is mediated by the leukocyte-specific collagen receptor LAIR-1, which suppresses lymphocytic activity through SHP-1 signaling (2). In addition, research shows that LAIR-1–induced immune suppression upon binding to its collagen ligand can be reversed by a decoy receptor LAIR-2 which has a higher binding affinity than LAIR-1 (3). Understanding the cross-talk between LAIR-1, LAIR-2, and associated ligands warrants further investigation for the development of novel therapeutic agents to overcome evasion of the immune system by some tumors.

Lung cancer is the leading cause of cancer-associated deaths worldwide due to late-stage disease presentation, metastasis, and resistance to therapies (4). The clinical implementation of immune checkpoint inhibitors (ICI) such as PD-1 or PD-L1 blockade has shown great improvement in the management of lung cancer (5). Despite the efficacy, development of acquired resistance to therapy has been reported in many patients with initial response (6). Recently, it was reported that PD-1 inhibitor–treated patients with increased collagen and LAIR-1 expression exhibit poorer response and survival outcomes in lung cancers (5, 7). The association of LAIR-1 with poor outcome in immunotherapy-treated patients merits further studies to explore its role in tumor biology. Altogether, this evidence indicates LAIR-1 could be a predictive biomarker for tumors which are resistant to current immunotherapy strategies.

The complexity of the tumor microenvironment (TME) may likely preclude the use of a single predictive biomarker of response to immunotherapy. The main objective of this study was to investigate LAIR-1 protein expression using quantitative immunofluorescence (QIF) and to assess its prognostic value alone or in combination with PD-L1 in patients with early-stage non–small cell lung cancer (NSCLC). Furthermore, using multiplexed QIF (mQIF), we investigated LAIR-1 protein expression across different subtypes of NSCLC and identified its localization in various tumor and stromal cell types comprising the NSCLC TME. Understanding the relative abundance of tumor and stromal cells where LAIR-1 is expressed can be a predictive feature to guide therapeutic decision-making through composite assessment of biomarkers and/or treatment regimens.

## Materials and Methods

### Patient Cohort and Tissue Microarray Construction

Formalin-fixed, paraffin-embedded (FFPE) tumor specimens represented in a tissue microarray (TMA) with NSCLC from two independent cohorts were analyzed in this study. First, multitumor TMA, YTMA-395, containing 295 cores from 12 different types of solid tumors and hematologic malignancies was tested to identify the predominant expression of LAIR-1 in different tumor types. A small lung test array, YTMA-295 containing 35 lung tumor cores was used to test four different anti-LAIR-1 antibody clones targeting nonoverlapping epitopes. The discovery cohort, Yale TMA (YTMA-423), contains the tissue specimens from 287 patients with early-stage NSCLC with tumors resected between 2011 and 2016. Table 1 shows the clinicopathologic characteristics of the patients included in YTMA-423. Patients with incomplete clinical information and insufficient tissue were excluded from the study. A total of 247 patients from the discovery cohort were analyzed. The validation cohort, YTMA-250, contains the tissue specimens from 144 patients. The clinicopathologic characteristics of YTMA-250 were shown in Supplementary Table S1. The size of each core within each TMA was 0.6 mm in diameter and represented an individual patient. Written informed consent, or waiver of consent, was obtained from all patients. All tissue samples were collected with approval from the Yale Human Investigation Committee protocol #9505008219.

**TABLE 1:** Clinicopathologic characteristics of NSCLC discovery cohort (YTMA-423)

Characteristic	Categories	*N* (%)	Total (%)
Age	68 (38–89)	246 (99.6)	246 (99.6)
Gender	Female	155 (62.8)	247 (100)
	Male	92 (37.2)	
Race	Asian	3 (1.2)	247 (100)
	Black	14 (5.7)	
	Other	1 (0.4)	
	White	229 (92.7)	
Smoking status	Current smoker	59 (23.9)	247 (100)
	Former smoker	154 (62.3)	
	Never smoker	34 (13.8)	
AJCC pathologic stage	IA	123 (49.8)	247 (100)
	IB	56 (22.7)	
	IIA	32 (13.0)	
	IIB	21 (8.5)	
	IIIA	14 (5.7)	
	IV	1 (0.4)	
Chemotherapy	Unknown	2 (0.8)	247 (100)
	No	195 (78.9)	
	Yes – Adjuvant after complete resection	45 (18.2)	
	Yes – Definitive regimen, + residual tumor	1 (0.4)	
	Yes – Neoadjuvant (prior to resection)	4 (1.6)	
Family cancer history	Unknown	1 (0.4)	247 (100)
	No	86 (34.8)	
	Yes	160 (64.8)	
Surgery	No	0 (0)	247 (100)
	Yes	247 (100)	
Histology	Squamous	57 (23.1)	247 (100)
	Adenocarcinoma	181 (73.3)	
	Other	9 (3.6)	

### Single and Multiplex Immunofluorescence Staining on TMA Serial Sections

Quantitative titration of four monoclonal LAIR-1 antibody clones including E7X6I (Cell Signaling Technology), NKTA25 (Santa Cruz Biotechnology), 1A10 (NC; NextCure), and 1E4 (NC) were performed. The optimized final concentration for each antibody, using the standard approach in our lab (8), was 0.25 μg/mL for E7X6I, 1 μg/mL for NKTA25, 0.25 μg/mL for 1A10, and 0.05 μg/mL for 1E4, respectively. For simultaneous detection of markers by isotype-specific antibodies, 5-plex or 4-plex immunofluorescence staining was performed on FFPE serial tissue sections of the discovery cohort. Three clones including NKT25, 1A10 and 1E4 were tested earlier in the experiment hence the serial TMA sections and E7X6I was tested in a TMA a few sections away from the earlier tested TMAs at the later stage of the experiment. The antibodies used for multiplexing and their optimized titrations were CD34 (1:4500; clone: QBE10, Dako), CD56 (1:200; clone: 123C3, Cell Signaling Technology), CD66b (1:500; clone: 80H3, LifeSpan Biosciences), CD8 (1:250; clone: 144B, Dako), CD14 (1:500; clone: D7A2T, Cell Signaling Technology), CD45 (1:200; clone: 2B11 + PD7/26, Dako), CD68 (1:200; clone: C8/144B, Dako), CD20 (1:150; clone: L26, Dako), CD4 (1:100; clone: SP35, SpringBio), SMA (1:500; clone: 1A4, Dako), CD163 (1:7500; clone: 10D6, Leica), CD11b (1:250; clone: CL1719, Novus), CK (cytokeratin; 1:100, clone: AE1AE3, Dako), and PD-L1 (1.1 μg/mL; clone: E1L3N, Cell Signaling Technology) were tested in eight multiplex panels including: (i) LAIR-1/CD45/CD14/DAPI, (ii) LAIR-1/CD4/CD8/CK/DAPI, (iii) LAIR-1/CD68/CD163/CK/DAPI, (iv) LAIR-1/CD163/CD56/DAPI, (v) LAIR-1/CD66B/SMA/DAPI, (vi) LAIR-1/CD11b/CD20/CD34/DAPI, (vii) LAIR-1/CD4/CK/DAPI, and (viii) PD-L1/CD68/CD163/CK/DAPI. The protocol for multiplexed immunofluorescence staining is detailed in the Supplementary Materials and Methods.

### Image and Data Analysis Using Two Independent Methods (QIF, and Cell Segmentation and Phenotyping)

First, the AQUA (automated quantitative analysis) method was used to measure the protein expression on fluorescence images acquired using a PM-2000 system (Navigate BioPharma) as described previously (9). A total compartment consisting of all cells was generated by thresholding the DAPI signal. The staining of CK defined the tumor compartment, and the stromal compartment was created by excluding the tumor compartment. A QIF AQUA score for each protein's expression was then measured by dividing the sum of target pixel intensities by the area of the designated compartment. Second, the pattern recognition software, inForm Tissue Finder 2.4.9 (Akoya Biosciences), was run on multispectral images acquired using Vectra/Polaris (Akoya) microscope. Cell segmentation within tumor and stroma regions was performed using the signals of the nuclei, cytoplasm, and membrane components as individual cells. Cell phenotyping was performed once the machine learning cell segmentation algorithm was optimized. Cells identified with LAIR-1 positivity on various cell types such as CK (epithelial cells), SMA (fibroblasts), CD34 (hematopoietic progenitor cells), CD14, CD68, CD163 (macrophages), CD45, CD4, CD8, CD20, CD11b, CD56 (natural killer, NK cells) and CD66b (neutrophils) were counted. Finally, the cell counts for each phenotype as cell densities/area (mm^2^) were analyzed using phenoptrReports (Akoya Biosciences) in R.

### Gene Expression Analysis from Bulk RNA sequencing and Single-cell RNA-sequencing Data

RNA sequencing (RNA-seq) data and the clinical metadata in read counts of nine human tumors were obtained from The Cancer Genome Atlas (TCGA) data portal (https://portal.gdc.cancer.gov/). The clinical information was filtered to exclude the data from solid tissue normal, and only the samples from the primary tumor and metastatic samples were analyzed. *LAIR-1* mean RNA expression across all tumor types was compared to select *LAIR-1* highly expressing tumors for further analyses. For validation, single-cell RNA-seq data from three lung carcinomas were downloaded from https://www.ncbi.nlm.nih.gov/geo/query/acc.cgi?acc=GSE127465 (10) to determine the expression of *LAIR-1* on specific cell types.

### Statistical Analysis

Statistical analyses were performed using GraphPad Prism 9.2.0 (GraphPad Software Inc.) and RStudio 2022.12.0+353 (2009–2022 Posit Software, PBC). Comparison between two continuous variables was assessed for linear association using Pearson correlation coefficient. The unpaired *t* test or one-way ANOVA followed by Tukey test for multiple comparisons as appropriate was used to compare the means between two or more groups, respectively. For the survival analysis, the patients were split into high and low LAIR-1 expression or LAIR-1 + cell phenotypes using the visual limit of detection as a cutoff. Kaplan–Meier plots for OS was computed, and comparisons were made by the log-rank test using survival and survminer packages in R studio. All statistical tests were two sided, and significance was represented as (*) *P* < 0.05 and (**) *P* < 0.01.

### Data Availability Statement

RNA-seq data and the clinical metadata in read counts of nine human tumors and single-cell RNA-seq data from three lung carcinomas were obtained from https://portal.gdc.cancer.gov/ and https://www.ncbi.nlm.nih.gov/geo/query/acc.cgi?acc=GSE127465 (10), respectively. Raw images supporting the findings of this study were submitted to https://www.ebi.ac.uk/bioimage-archive with the accession number TMP_1659384798859.

## Results

### LAIR-1 Antibody Validation and Optimization

**FIGURE 1:**
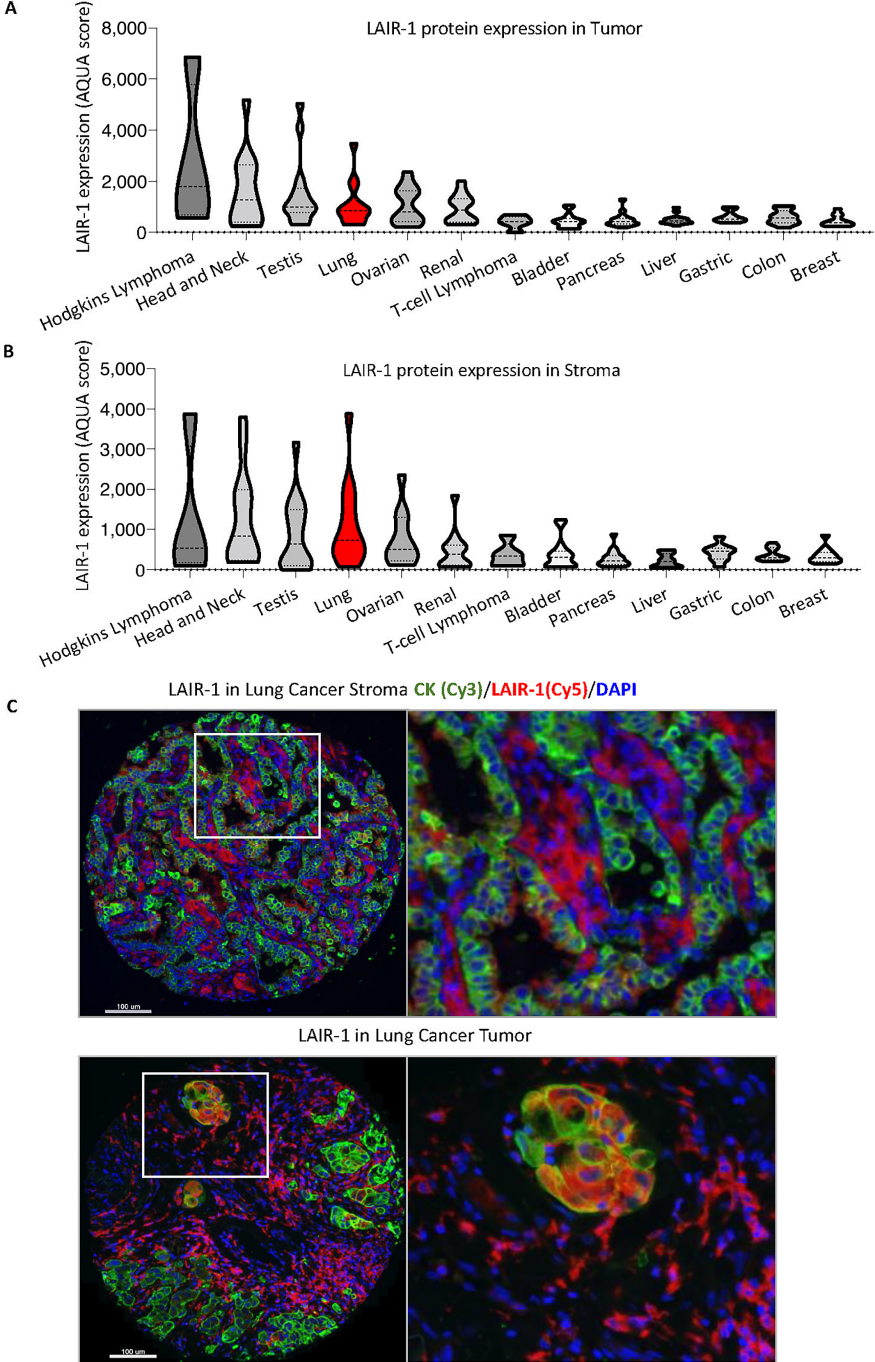
Expression of LAIR-1 in multitissues LAIR-1 tumor (**A**) and stromal (**B**) protein expression in multiple tissues. **C,** Representative images of LAIR-1 stromal and tumor expression in lung cancer. CK: green, LAIR-1: red, DAPI: blue. The fluorescent channels where each marker was acquired were shown in brackets.

Gene expression analysis from bulk RNA-seq of nine tumor types obtained from TCGA (https://portal.gdc.cancer.gov/repository) showed that *LAIR-1* is highly expressed in both LUAD and lung squamous cell carcinoma (LUSC; Supplementary Fig. S1A). The protein expression pattern of LAIR-1 in multitumor TMA (YTMA-395) from 12 different types of solid tumors and hematologic malignancies showed that LAIR-1 is more highly expressed in lung cancer (Fig. 1A and B) compared with most other tumor types. We then tested four different anti-LAIR-1 antibody clones targeting nonoverlapping epitopes in a lung test array (YTMA-295) containing 35 lung tumor cores (Supplementary Fig. S1B–E and S2A–D). We observed a specific LAIR-1 staining pattern localized predominantly in the plasma membrane with all antibodies. We then compared the LAIR-1 AQUA scores between four clones in the discovery cohort containing 247 patients with NSCLC. A high correlation coefficient was observed between the clones 1A10 and 1E4 (*R* = 0.77; Supplementary Fig. S1F). Both mAbs bind to the intracellular domains of LAIR-1 which are LAIR-1A and LAIR-1B. 1E4 clone has higher binding affinity than 1A10. The staining quantification of the four LAIR-1 clones was shown in Supplementary Fig. S2. The concordant staining pattern of 1E4 and 1A10 was observed and the highest staining pattern of 1E4 among all four clones with the lowest signal/noise ratio was noted. As a result, LAIR-1 expression measurement with the 1E4 clone showed good reproducibility between two independent experiments (*R* = 0.82; Supplementary Fig. S1G). Therefore, we considered the clone 1E4 validated and used it for the subsequent experiments in this study.

### LAIR-1 Expression in Both Tumor and Stromal Cells of NSCLC Cohorts

**FIGURE 2:**
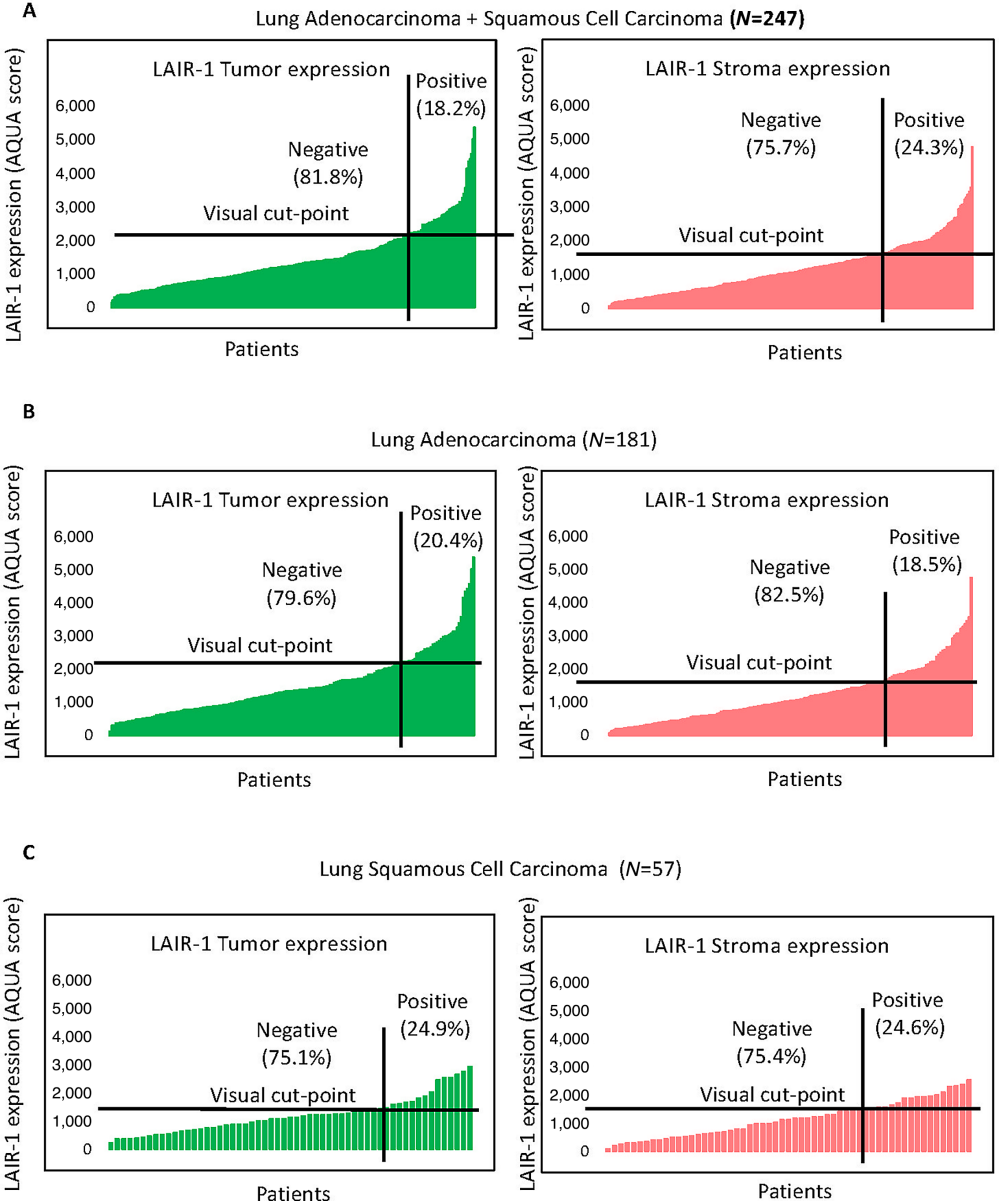
Expression of LAIR-1 in lung tumor tissues. **A,** LAIR-1 tumor and stromal expression in NSCLC discovery cohort (LUAD+LUSC, *N* = 247). **B,** LAIR-1 tumor and stromal expression in patients with LUAD of lung discovery cohort (*N* = 181). **C,** LAIR-1 tumor and stromal expression in patients with LUSC of lung discovery cohort (*N* = 57).

The expression pattern of LAIR-1 in human NSCLC was detected in both tumor and stromal cells (Fig. 1C). Consistently, *LAIR-1* RNA expression in single-cell sequencing of seven lung carcinomas confirmed its expression in several stromal immune cells (Supplementary Fig. S3A and S3B; ref. 10). Tumor and stroma LAIR-1 expression were observed in both the discovery and validation cohorts (Fig. 2; Supplementary Fig. S4A). Our finding of LAIR-1 stromal expression is consistent with the report of Meyaard and colleagues that LAIR-1 is expressed on many immune cells, including T cells, B cells, NK cells, monocytes, and dendritic cells (1). In the stroma, approximately 24% was visually positive for LAIR-1. A fair correlation between LAIR-1 tumor and stromal expression (Pearson *R* = 0.74) in the discovery cohort was noted, confirming its expression in both compartments (Supplementary Fig. S1H). When we performed the staining, we assessed the cell line control array (the positive and negative indicator for LAIR-1 expression) in parallel. We determine the threshold of negativity based on LAIR-1–negative cell lines and any staining artifacts in tumor cores below the threshold were determined negative. Any tumor cores that are above the threshold were visually evaluated to determine true positivity and cases with less than 1% tumor (CK staining) were excluded from the analyses. According to the visually defined cutoff that splits the NSCLC cases into high/positive versus low/negative expression (signal vs. background noise), tumor LAIR-1 positivity was detected in approximately 18% of patients with NSCLC. The notion that LAIR-1 is highly expressed on tumor cells requires further evaluation on tumor cell intrinsic function of LAIR-1 in the context of therapeutic blockade of tumor LAIR-1 versus immune LAIR-1. In the discovery cohort, approximately 73% of the cases are adenocarcinoma, approximately 23% are squamous cell carcinoma and approximately 4% of the cases are other subtypes. (Table 1). In the validation cohort, approximately 58% of the cases are adenocarcinoma, approximately 25% of the cases are squamous cell carcinoma and the remaining cases belong to other subtypes (Table 2). The expression pattern of LAIR-1 in NSCLC subtypes is consistent across both cohorts (Fig. 2B and C; Supplementary Fig. S4A). These results led us to investigate the distribution of LAIR-1 expression in both immune and tumor cells and its prognostic value.

**TABLE 2:** Univariable and multivariable Cox proportional hazards regression analyses to assess the association of LAIR-1 expression in LUAD tumor subtypes (*N* = 181) from NSCLC cohort 1 and the clinical pathologic features regarding OS

	Univariable analysis		Multivariable analysis	
Variables	HR (95% CI)	*P*	HR (95% CI)	*P*
LAIR-1 in Tumor (LUAD)	2.4 (1.2–4.9)	0.022*	2.3 (1.14–6.03)	0.024*
Age	1.0 (0.99–1.1)	0.14	1.07 (1.01–1.12)	0.012*
Race	0.68 (0.32–1.4)	0.3	0.78 (0.2–3.05)	0.72
Gender (M vs. F)	1.3 (0.62–2.7)	0.49	1.46 (0.61–3.48)	0.4
Stage (vs. I)				
II	3.2 (1.3–7.5)	0.009**	3.86 (1.5–9.8)	0.005**
III	9.6 (4.1–22.2)	0.001***	12.86 (4.3–38.9)	0.001***
IV	9.0 (0.96–53.8)	0.035	10.78 (1.19–97.9)	0.035*
Smoking status at enrollment (vs. current smoker)				
Former smoker	0.53 (0.25–1.14)	0.11	0.44 (0.16–1.16)	0.098
Never smoker	0.15 (0.07–0.82)	0.013*	0.13 (0.025–0.64)	0.013*
Family cancer history (No vs. Yes)	0.45 (0.23–0.9)	0.025*	0.59 (0.26–1.33)	0.201

Statistical significance was represented as (*) *P* < 0.05, (**) *P* < 0.01, and (***) *P* < 0.001.

### LAIR-1 Expression in Two Major Cell Types; Tumor Cells and Macrophages, in NSCLC Cohort

**FIGURE 3:**
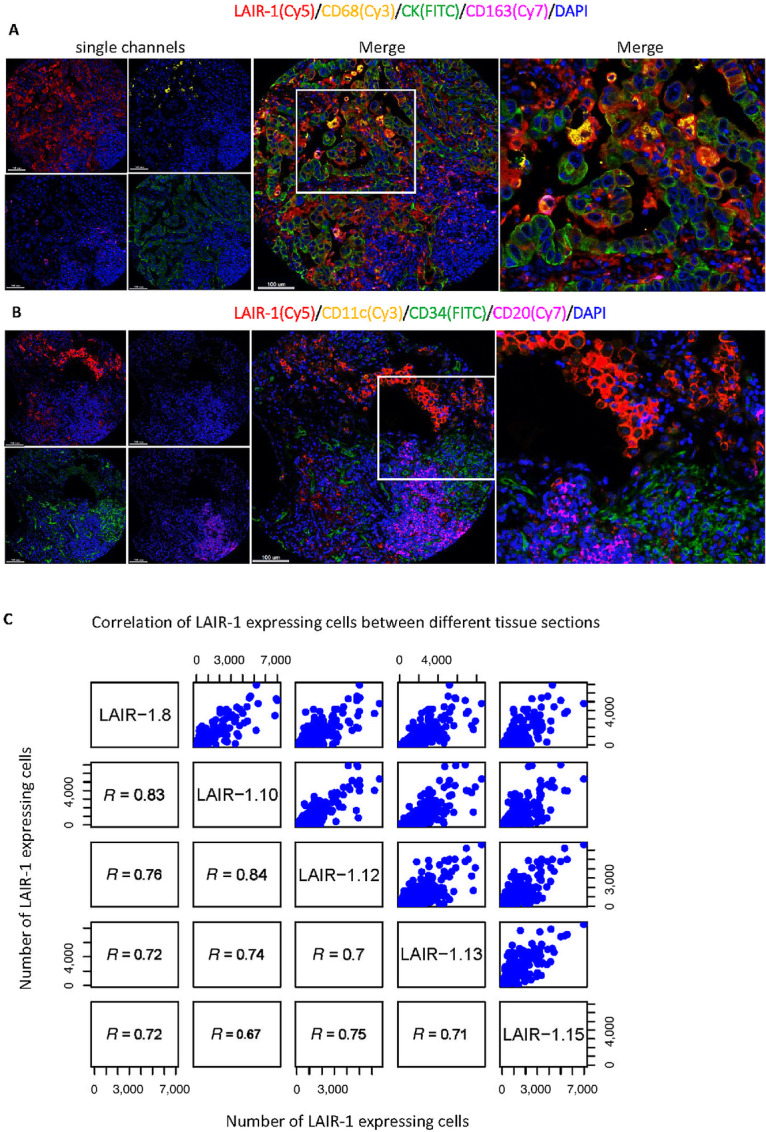
Identification of LAIR-1 expression in various cell types. Representative mQIF images of 5-plex panels (**A**) and LAIR-1/CD11c/CD34/CD20/DAPI (**B**). The fluorescent channels where each marker was acquired were shown in brackets. **C,** Correlation of LAIR-1–expressing CK^+^ tumor cells between different sections.

**FIGURE 4:**
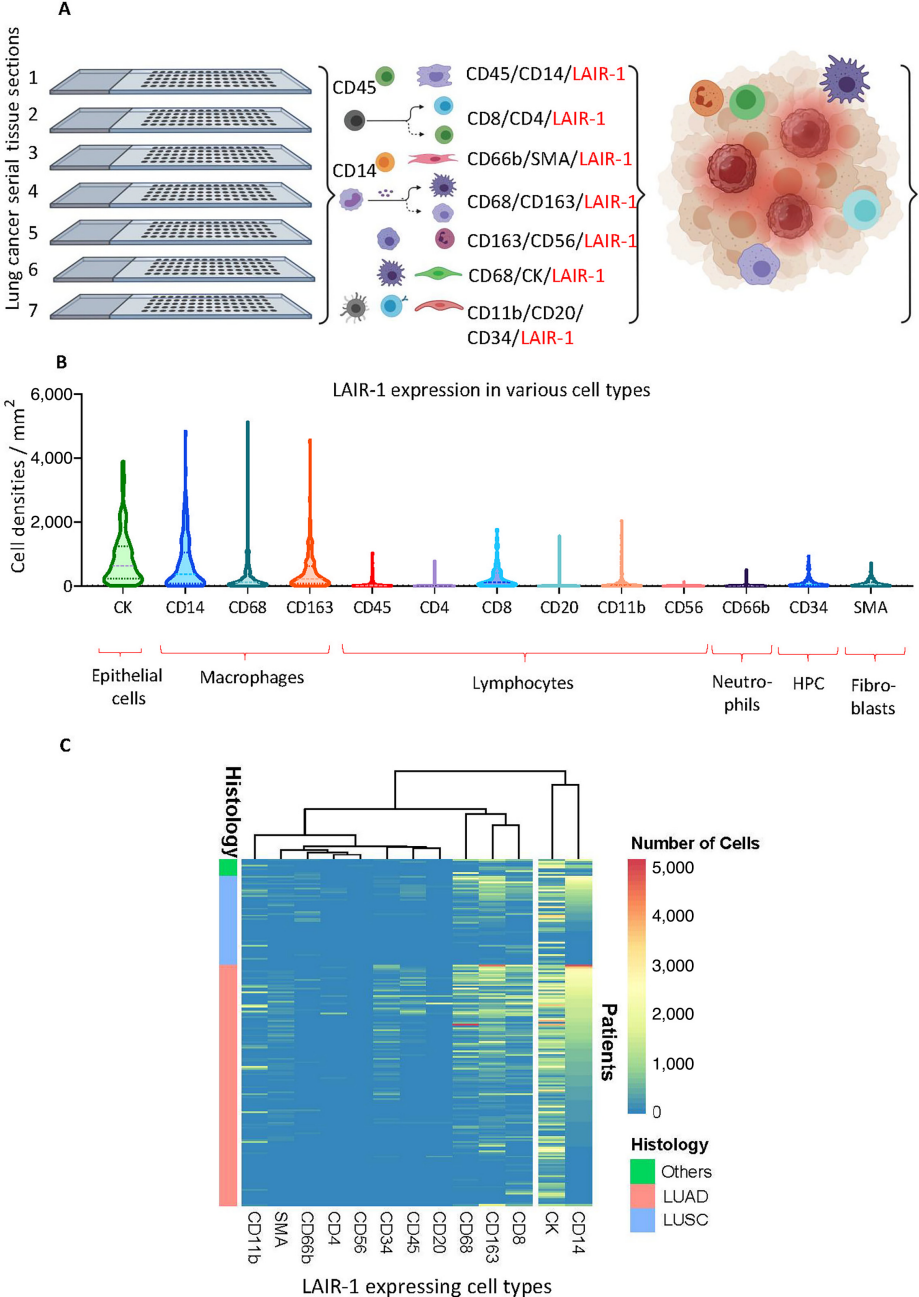
Quantification of LAIR-1–expressing cell densities/mm^2^ within both stroma and tumor. **A,** Schematic diagram demonstrating the identification of various cell types expressing LAIR-1 on mQIF from Lung TMA serial sections (created with BioRender.com). **B,** Quantification of LAIR-1–expressing cell densities/mm^2^ in Lung TMA serial tissue sections. **C,** Heatmap illustrating the abundance of LAIR-1–expressing cell types in different histologic subtypes of NSCLC.

The distribution of LAIR-1 expression on various cell types were identified by multiplexed QIF on serial sections of the discovery cohort. However, often protein markers were only partially accessed when staining the tissues due mainly to the limitations of isotype-specific antibodies and cross-reactivity that obscures overlap of fluorescent spectra. These limitations represent challenges for measurement of characteristics such as coexpression and spatial relationships. In addition, autofluorescence of FFPE tissue sections further complicates the interpretation and quantitative analysis. As such, parallel evaluation of the prognostic and predictive value of biomarkers expressing various cell types has been limited. Here, we used a multiplexed IHC technique coupled with simultaneous imaging readout and advanced trainable pattern recognition software to visualize tissue regions (i.e., tumor, stroma, and background) and quantify the composition of tumor and the TME cells (cells densities/mm^2^) colocalized with LAIR-1 in serial TMA sections (Fig. 4A). LAIR-1 was multiplexed with lymphocyte markers (CD45RA, CD4, CD8, CD20, CD56), myeloid monocyte markers (CD14, CD68, CD163 for macrophages and CD66b for neutrophils), fibroblast marker (SMA), hematopoietic progenitor cells (HPC, CD34) and tumor marker (pan CK). Representative multispectral QIF images of 5-plex panels are shown in Fig. 3A and B. The correlation coefficient between LAIR-1 expression of serial TMA sections showed high reproducibility of IHC multiplexed assays ranging from Pearson *R* = 0.67 to *R* = 0.84 (Fig. 3C).

It has been reported that high expression of LAIR-1 is observed on CD4^+^ T cells (70%–80%), CD8^+^ T cells (80%–90%), CD56^+^ NK cells (95%–100%), CD19^+^ B cells (80%–90%), and CD14^+^ monocytes (99%–100%; refs. 1, 11). To further subtype immune cell populations, QIF staining of a multiplexed IHC panel including LAIR-1, CD45RA, and CD14 was performed. LAIR-1 was observed to be expressed on pan CK^+^ tumor cells and CD14^+^ monocytes. Within monocytes, the macrophages, defined by CD68 and the alternatively activated M2 macrophages defined by CD163 showed prominent LAIR-1 expression, whereas weak LAIR-1 expression was observed on CD66b^+^ neutrophils and CD11c^+^ dendritic cells (Fig. 4B and C). However, within lymphocyte markers, low expression of LAIR-1 was observed on CD4^+^ T cells, CD20^+^ B cells, CD11b^+^ dendritic cells, and CD56^+^ NK cells except on CD8^+^ T cells where moderately high LAIR-1 expression was noted (Fig. 4C). Our results from the NSCLC discovery cohort were validated by single-cell sequencing of three lung carcinomas (10) where LAIR-1 was highly expressed on CK8/CK10+ tumor cells and CD68^+^ and CD163^+^ macrophages (Supplementary Fig. S3A and S3B). These data suggests that tumor cells and macrophages are the two types of cells that most highly express LAIR-1.

### Prognostic Value of LAIR-1 in Tumor Cells and Macrophages

**FIGURE 5:**
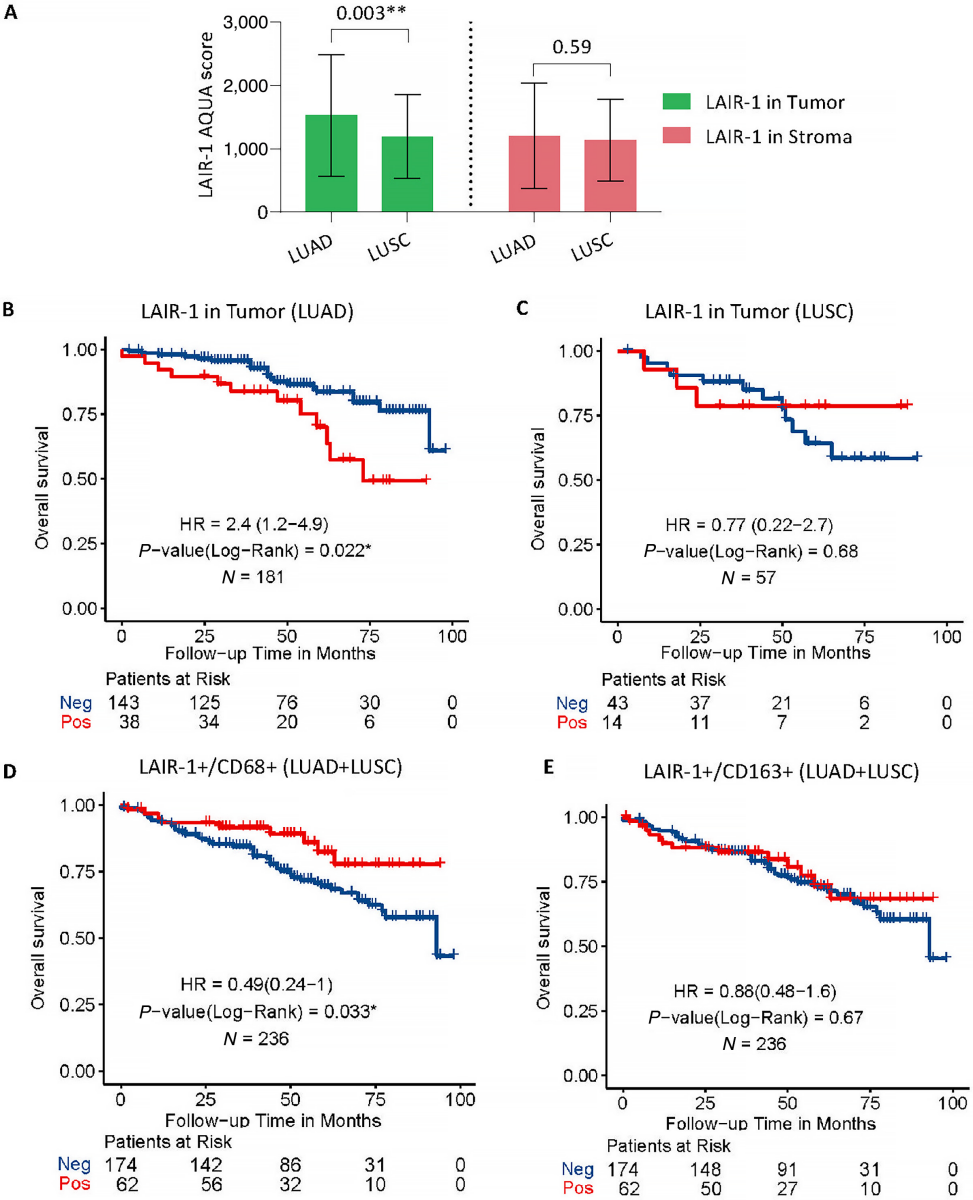
Identification of LAIR-1 expression in different molecular compartments associated with OS in the discovery cohort. **A,** Bar plot showing the difference in LAIR-1 expression within tumor and stromal compartments of two NSCLC histologic. **B,** Kaplan–Meier plot identifying the tumor LAIR-1 expression and its association with OS in LUAD subtype. **C,** Kaplan–Meier plot identifying the tumor LAIR-1 expression and its association with OS in LUSC subtype. **D,** Kaplan–Meier plot identifying the association of LAIR-1–expressing CD68 cells with OS in the discovery cohort. **E,** Kaplan–Meier plot showing the association of LAIR-1–expressing CD163 cells with OS in the discovery cohort. Statistical significance was represented as *, *P* < 0.05 and **, *P* < 0.01.

In our study, the mean LAIR-1 tumor expression level of adenocarcinoma patients is higher than that of patients with squamous cell carcinoma (**, *P* = 0.003) but not the mean stromal expression (*P* = 0.59) in the discovery cohort (Fig. 5A). A similar trend for the mean LAIR-1 expression of both tumor (*P* = 0.06) and stroma (*P* = 0.09) was observed in the validation cohort (Supplementary Fig. S4A and S4B). It has been reported that increased LAIR-1 expression is associated with worse outcomes in patients treated with immunotherapy (5, 12). To also investigate a potentially prognostic significance, we assessed the association of LAIR-1 expression with survival in the whole NSCLC discovery cohort (i.e., LUAD + LUSC+ other), as well as individually in adenocarcinoma (LUAD) and squamous cell carcinoma (LUSC) patient subgroups. As a cut-off point, we used the visual limit of detection which defines approximately 18% of the population as positive for LAIR-1 (Fig. 2A). Neither tumor (*P* = 0.56) nor stromal (*P* = 0.93) LAIR-1 expression in the whole discovery cohort significantly predicted OS. Similarly, neither stromal nor tumor LAIR-1 expression of patients with LUSC histologic subtype significantly predicted OS after surgery. In contrast, high LAIR-1 tumor expression in LUAD subtype was significantly associated with reduced OS (HR = 2.4; *, *P* = 0.022; Fig. 5B). Similarly, we observed the association of tumor but not stromal LAIR-1 expression with OS in the LUAD subtype of the validation cohort as well (HR = 2; *, *P* = 0.05; Supplementary Fig. S4C). Stromal LAIR-1 expression in patients with LUAD did not significantly affect event risk. This might be due to the composition of the heterogenous population of LAIR-1–expressing immune cells and different molecular subtypes of cells within the stroma. Multivariate assessment of the association between LAIR-1 expression in LUAD subtype of the discovery cohort and clinicopathologic characteristics demonstrated that LAIR-1 expression in NSCLC is associated with age, smoking status, and pathologic stage (Table 2).

Consequently, we assessed the association of LAIR-1 expression with the cell densities of LAIR-1^+^/CD68^+^ M1/M2 and LAIR-1^+^/CD163^+^ M2 macrophage because CD14^+^, CD68^+^, and CD163^+^ cells predominantly express LAIR-1 (Fig. 5D and E). High number of CD68^+^ cells expressing LAIR-1 in the discovery cohort shows positive correlation with the outcome (HR = 0.49; *, *P* = 0.033; Fig. 5D). No association of LAIR-1^+^/CD163^+^ cells with OS was detected. We additionally assessed the cell densities of LAIR-1^+^/CK^+^ tumor cells with regards to their association with OS as compared with LAIR-1 QIF scores in tumor compartment by AQUA. Our results indicated that high densities of LAIR-1^+^/CD68^+^ cells improved the outcome (HR = 0.49; *, *P* = 0.033) in the whole NSCLC discovery cohort (LUAD + LUSC + other). Consistent with the finding of tumor compartmentalized AQUA scores, patients with high LAIR-1^+^/CK^+^ tumor cell densities showed worse outcomes (HR = 1.5) in both discovery and validation cohorts (Supplementary Fig. S4C). Taken together, our results indicated that LAIR-1 is highly expressed in LUAD subtype, and that high level of LAIR-1 expression is associated with poor survival, suggesting LAIR-1 in tumor can be an independent prognostic marker for patients with LUAD subtype.

### LAIR-1 and PD-L1 Expression in Patients with NSCLC

**FIGURE 6:**
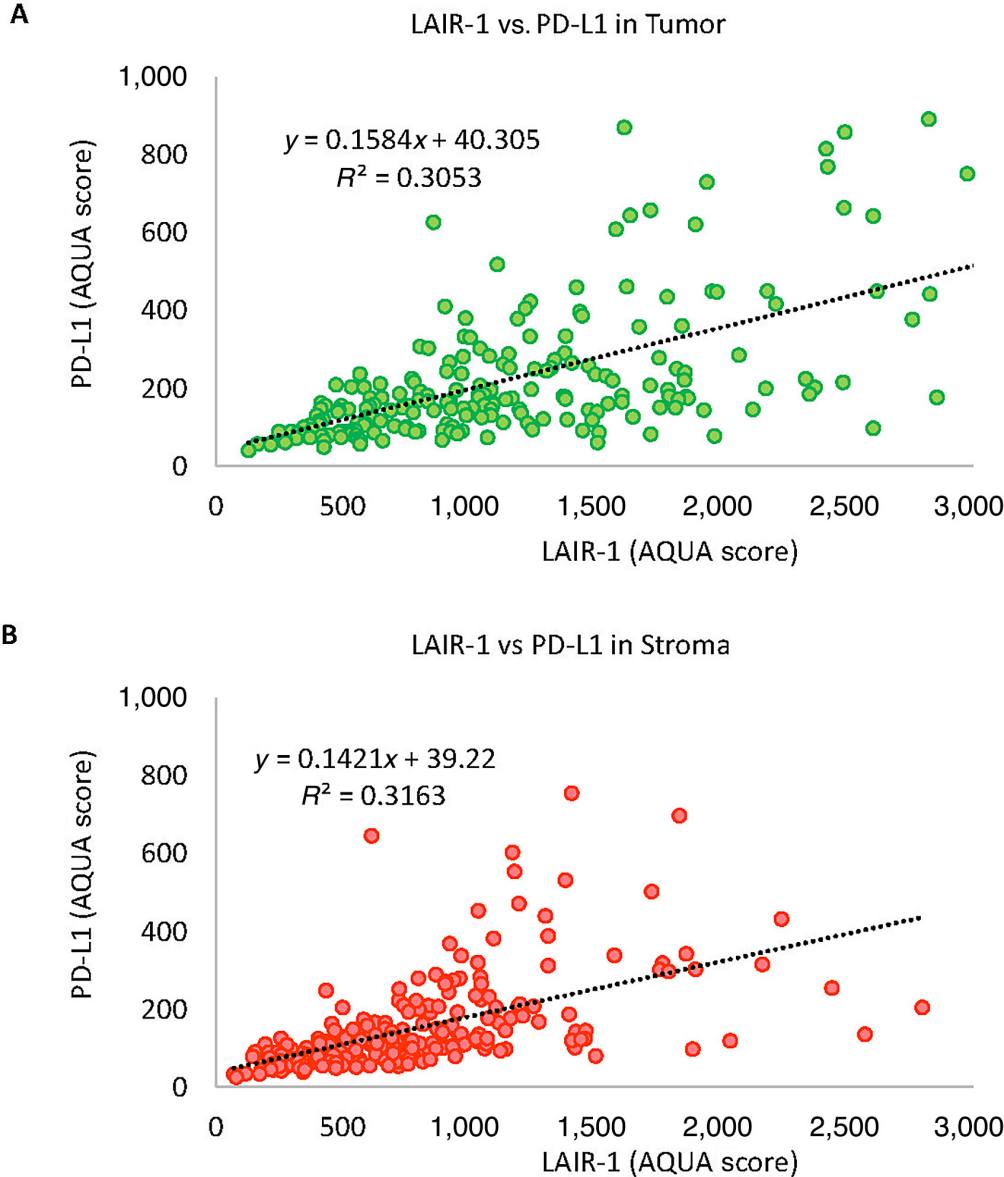
Correlation between LAIR-1 and PD-L1 expression. Regression coefficient (*R*^2^) between LAIR-1 and PD-L1 expressing within tumor (**A**) and stromal (**B**) compartments.

Targeting LAIR-1 through the inhibitory protein tyrosine phosphatase SHP-1 signaling could sensitize tumors resistant to PD-1/PD-L1 axis blockade which leads to the reduction of tumor growth and metastasis in the mouse model (5). To evaluate the clinical relevance of the correlation either synergistically or independently between LAIR-1 and PD-L1 expression, we assessed the expression of both proteins multiplexed with CD68, CD163, and CK in two serial TMA sections. We observed a weak correlation between tumor LAIR-1 and tumor PD-L1 expression (*R*^2^ = 0. 31; Fig. 6A) and stromal LAIR-1 and stromal PD-L1 expression (*R*^2^ = 0.32; Fig. 6B). Several cases that were positive for LAIR-1 but negative for PD-L1 were noted. In previous studies, LAIR-1 expression was mainly reported to be in immune cells rather than tumor cells. The correlation of LAIR-1 and PD-1 in addition to PD-L1 could provide valuable insight into clinical relevance of LAIR-1 expression and blockade of ITIM signaling.

## Discussion

The interactions between the ITIM containing receptors and their ligands including but not limited to PD-1/PD-L1 and CTLA-4/CD80/86, are known to be involved in tumor development as well as loss of immune regulation. Targeting these receptors with therapeutic ICIs has shown outstanding achievement in cancer management (13). ICI, either alone or in combination regimens, has become the standard of care in the treatment of several advanced malignancies. Here, we discuss an ITIM containing checkpoint LAIR-1, also known to suppress the function of several immune cell subsets upon binding to its ligand (1, 14–16). This study shows that LAIR-1 is highly expressed in Hodgkin lymphoma, head and neck, testicular, and lung cancer. We focused on LAIR-1 and its expression in various cell types within NSCLC. Consistent with previous findings (1, 11), we observed that LAIR-1 is expressed on several stromal and immune cells in patients with NSCLC. The widespread expression pattern of LAIR-1 across lung carcinomas especially in NSCLC invokes the need for its study in TME regulation.

NSCLC is a heterogeneous disease comprising several subtypes, of which LUAD and LUSC are the most frequent subtypes (17). Significantly higher tumor LAIR-1 expression of LUAD cases than that of LUSC cases was noted in the discovery cohort, although stromal LAIR-1 expression in both subtypes shows no difference. Our results were in agreement with previous reports suggesting that the tumor immune microenvironment (TIME) of LUAD and differs substantially LUSC (18). With regards to overall survival in both cohorts, we observed a significant association with high LAIR-1 tumor expression and worse outcome in LUAD cases and but not in LUSC cases. Conversely, high number of CD68 cells expressing LAIR-1 in the discovery cohort shows positive association with the outcome. Tumor-associated macrophages have been reported to be key attributers to inflammation in LUSC TIME, while regulatory B cells have shown immunosuppressive and tumorigenic functions in LUAD TIME (19). This indicates that the prevalence of immunosuppressive tumor-associated macrophages in LUAD subtype precludes the antitumor immune response and reduces the effectiveness of immune checkpoint blockades in LUAD subtype of patients with NSCLC. The fact that high expression of LAIR-1 in patients with LUAD is associated with worse OS in our study suggests that LAIR-1 can be potential alternative target for patients resistant to ICIs. Altogether this suggests that understanding the differences of LAIR-1 expression in TME between NSCLC subtypes may play an important role in identifying patients who will benefit the most from immunotherapy.

Because we noted the stromal LAIR-1 expression in our cohorts, we assessed different populations of stromal cells expressing LAIR-1 including immune cells, fibroblasts, and HPC. In line with results published previously (11, 20), we observed that monocytes characterized by CD14, and macrophages characterized by CD68 and CD163, predominantly express LAIR-1 compared with other cell types. Previous work suggested that LAIR-1 is highly expressed on CD14^+^ intermediate monocytes as compared with other monocytic subtypes (11), and CD68 is highly expressed on these intermediate monocytes (21). Under inflammatory conditions, LAIR-1 is upregulated in intermediate monocytes through IFNα-mediated response, although persistent inflammation is the main factor in the pathogenesis of many chronic diseases leading to a significant destruction of tissue and organs (11, 22, 23). Upon interacting with its ligand under inflammation, LAIR-1 becomes actively functional to dampen the immune responses (11). Recently, it was reported that the combination treatment of LAIR-1 inhibitor, NC410, and the blockade of TGFβ and PD-L1, ablated CD163^+^ M2 macrophage populations and modulates CD163^−^ M2 population toward a more M1-like phenotype leading to enhanced CD8^+^ T cells infiltration and repolarization of suppressive macrophage populations (24). These data and our results collectively suggest the importance of LAIR-1 targeting in restoring immune function.

In addition, we noted that LAIR-1 is highly expressed on tumor cells. This finding highlights the need for further evaluation regarding the therapeutic blockade options of tumor LAIR-1 versus immune LAIR-1. Because LAIR-1 expression is believed to be restricted to hematopoietic-derived cells in healthy conditions, the identification of LAIR-1 on tumor cells of nonhematopoietic origin indicates a deregulation of LAIR-1 expression in the TIME. Our study utilized a mAb specific for the intracellular region of LAIR-1, thus avoiding the potential for a LAIR-1 antibody to bind to cleaved LAIR-1 proteins tightly bound to LAIR-1–ligand interactions on cell membranes. As such, our study confirms expression of LAIR-1 on tumor cells, similar to expression of PD-1, rather than PD-L1 on tumor cells as described previously (25). The confirmation of LAIR-1 on tumor cells suggests further study of mechanisms of LAIR-1 expression on tumor cells, the role of tumor cell-intrinsic LAIR-1 signaling, and the role of LAIR-1 adhesion to tumor collagens is warranted.

There are a number of limitations to this work. Perhaps most significantly, this pilot effort was performed entirely on TMAs. While we often used more than one histospot per patient, we did not use whole tissue sections as are used for pathologist assessments of patient biopsies. The use of TMAs can limit the ability to assess the heterogeneity of expression. An additional challenge to this work is that LAIR-1 is a promiscuous receptor protein and can bind to a large number of ligands, including surfactant protein D (26), C1q (27), mannose binding lectin (28), and collagens (5). The differential effect of LAIR-1 interactions with each possible ligand cannot be easily addressed by our approach. Although we show expression of LAIR-1 in different cell types, we do not know whether the cell type–specific expression affects ligand preference and function. Finally, although other works have attributed immune checkpoint function to LAIR-1, we are unable to address that issue in this study.

In summary, previous data suggest that LAIR-1 might be targeted to normalize the function of the TIME. In our work, the range of LAIR-1 expression in both tumor subtypes and cell types shows its prognostic value in human NSCLC. Further efforts are required to validate our observations and to better understand the potential of LAIR-1 as an immune checkpoint target.

## Supplementary Material

Supplementary Figure FS1 Antibody Validation. (A) LAIR-1 mean RNA expression across nine tumor types. Representative QIF images of antibody validation using four different anti-LAIR-1 antibody clones (B) E7X61, (C) NKT25, (D) 1A10 and (E) 1E4 targeting non-overlapping epitopes in a lung test array (YTMA–295) containing 35 lung tumor cores. CK: green, LAIR-1: red, DAPI: blue. Three clones NKT25, 1A10 and 1E4 were tested earlier in the experiment hence the serial TMA sections and E7X61 was tested in a TMA a few sections away from the earlier tested TMAs later at the stage of the experiment. The fluorescent channels where each marker was acquired were shown in brackets. (F) Correlation of LAIR-1 protein expression scores measured by AQUA between four antibody clones. (G) Correlation plot showing the good reproducibility of staining by 1E4 clone between two independent experiments. (H) Correlation plot showing high expression LAIR-1 in both tumor and stroma.

Supplementary Table TS1 Clinicopathological characteristics of NSCLC validation cohort (YTMA-250).

Supplementary Figure FS2 Representative figures of staining by different antibody clones. Representative QIF images of different LAIR-1 clones staining targeting non-overlapping epitopes in a lung test array (YTMA–295) containing 35 lung tumor cores stained by (A) E7X61 clone, (B) NKT25 clone, (C) 1A10 clone and (D) 1E4 clone. (E) The staining quantification of the four LAIR-1 clones. CK: green, LAIR-1: red, DAPI: blue. The fluorescent channels where each marker was acquired were shown in brackets.

Supplementary Figure FS3 LAIR-1 expression in different molecular subtypes of within Storma and Tumor. (A) Umap (Uniform Manifold Approximation and Projection) plot showing the expression of LAIR-1 RNA in patient lung tumor biopsies (n=7) derived single cell RNA-seq data. (B) Quantification of LAIR-1 expression in LM22 immune cells showing highest expression of LAIR-1 in macrophages.

Supplementary Figure FS4 LAIR-1 expression in different compartments and its association with survival in Validation Cohort 250 (YTMA-250). (A) LAIR-1 protein expression in tumor and stroma compartments. (B) LAIR-1 tumor and stromal protein expression in both LUAD and LUSC-NSCLC subtypes. (C) Association of LAIR-1 expression in tumor and CD68 compartments with the outcome.
