# Supplementary figures and images for: Quantitative, Spatially Defined Expression of Leukocyte-associated Immunoglobulin-like Receptor in Non–small Cell Lung Cancer
Source: Cancer Res Commun. 2023 Mar 21;3(3):471–82. doi: 10.1158/2767-9764.CRC-22-0334 (PMC10029762; doi:10.1158/2767-9764.CRC-22-0334)

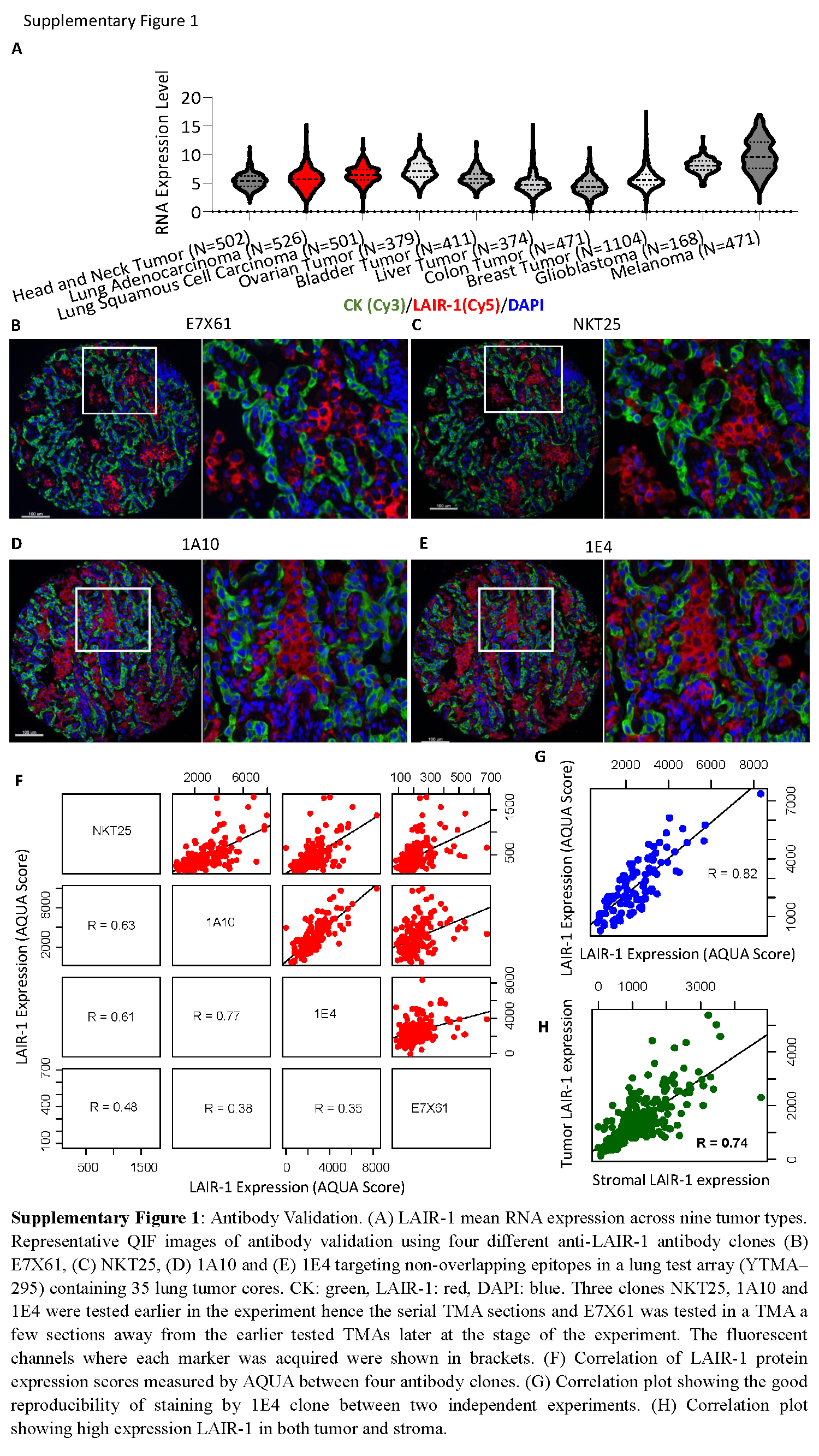

Supplement: Supplementary Figure FS1 — Antibody Validation. (A) LAIR-1 mean RNA expression across nine tumor types. Representative QIF images of antibody validation using four different anti-LAIR-1 antibody clones (B) E7X61, (C) NKT25, (D) 1A10 and (E) 1E4 targeting non-overlapping epitopes in a lung test array (YTMA–295) containing 35 lung tumor cores. CK: green, LAIR-1: red, DAPI: blue. Three clones NKT25, 1A10 and 1E4 were tested earlier in the experiment hence the serial TMA sections and E7X61 was tested in a TMA a few sections away from the earlier tested TMAs later at the stage of the experiment. The fluorescent channels where each marker was acquired were shown in brackets. (F) Correlation of LAIR-1 protein expression scores measured by AQUA between four antibody clones. (G) Correlation plot showing the good reproducibility of staining by 1E4 clone between two independent experiments. (H) Correlation plot showing high expression LAIR-1 in both tumor and stroma. [file crc-22-0334-s01.png]

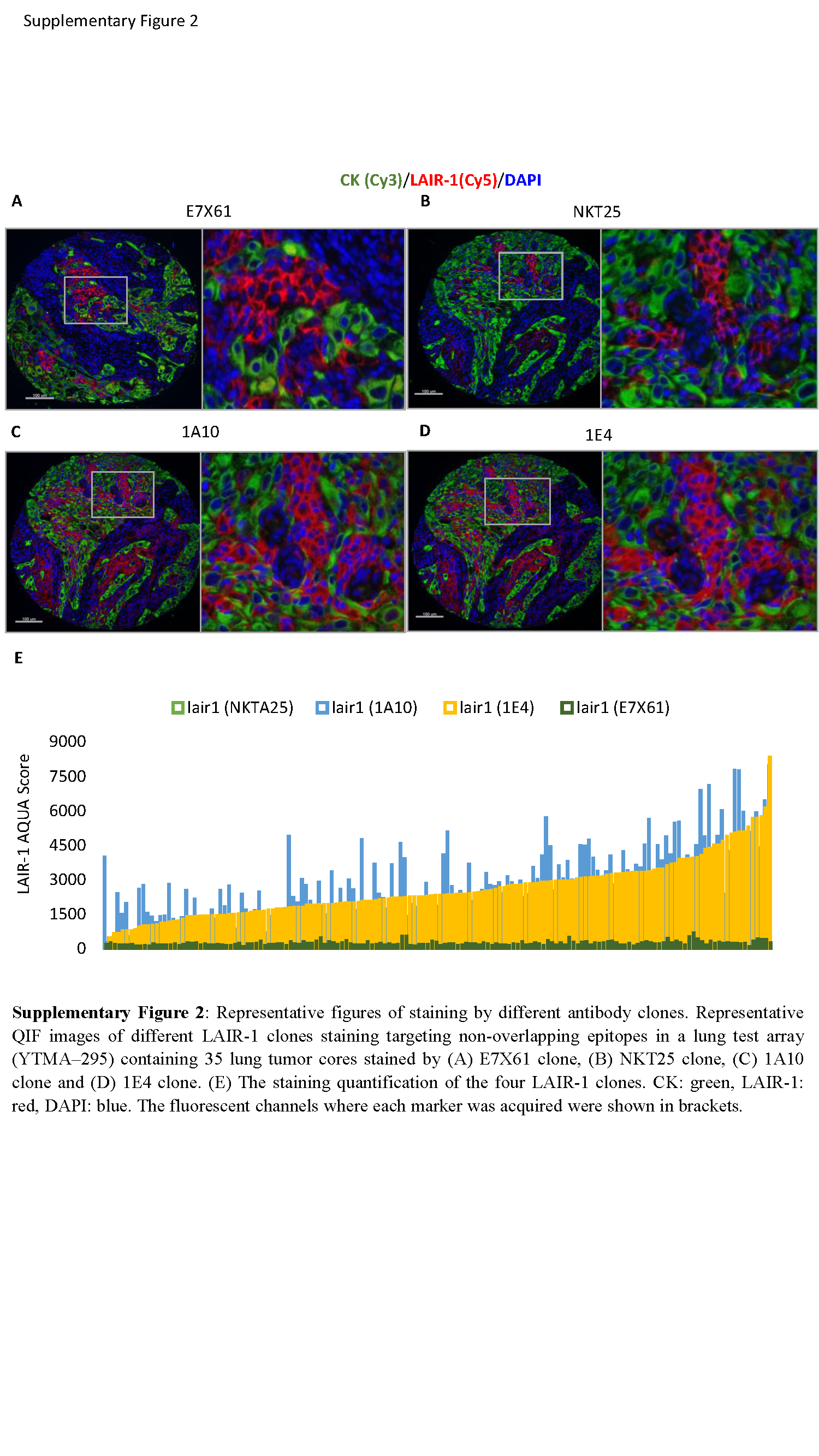

Supplement: Supplementary Figure FS2 — Representative figures of staining by different antibody clones. Representative QIF images of different LAIR-1 clones staining targeting non-overlapping epitopes in a lung test array (YTMA–295) containing 35 lung tumor cores stained by (A) E7X61 clone, (B) NKT25 clone, (C) 1A10 clone and (D) 1E4 clone. (E) The staining quantification of the four LAIR-1 clones. CK: green, LAIR-1: red, DAPI: blue. The fluorescent channels where each marker was acquired were shown in brackets. [file crc-22-0334-s03.png]

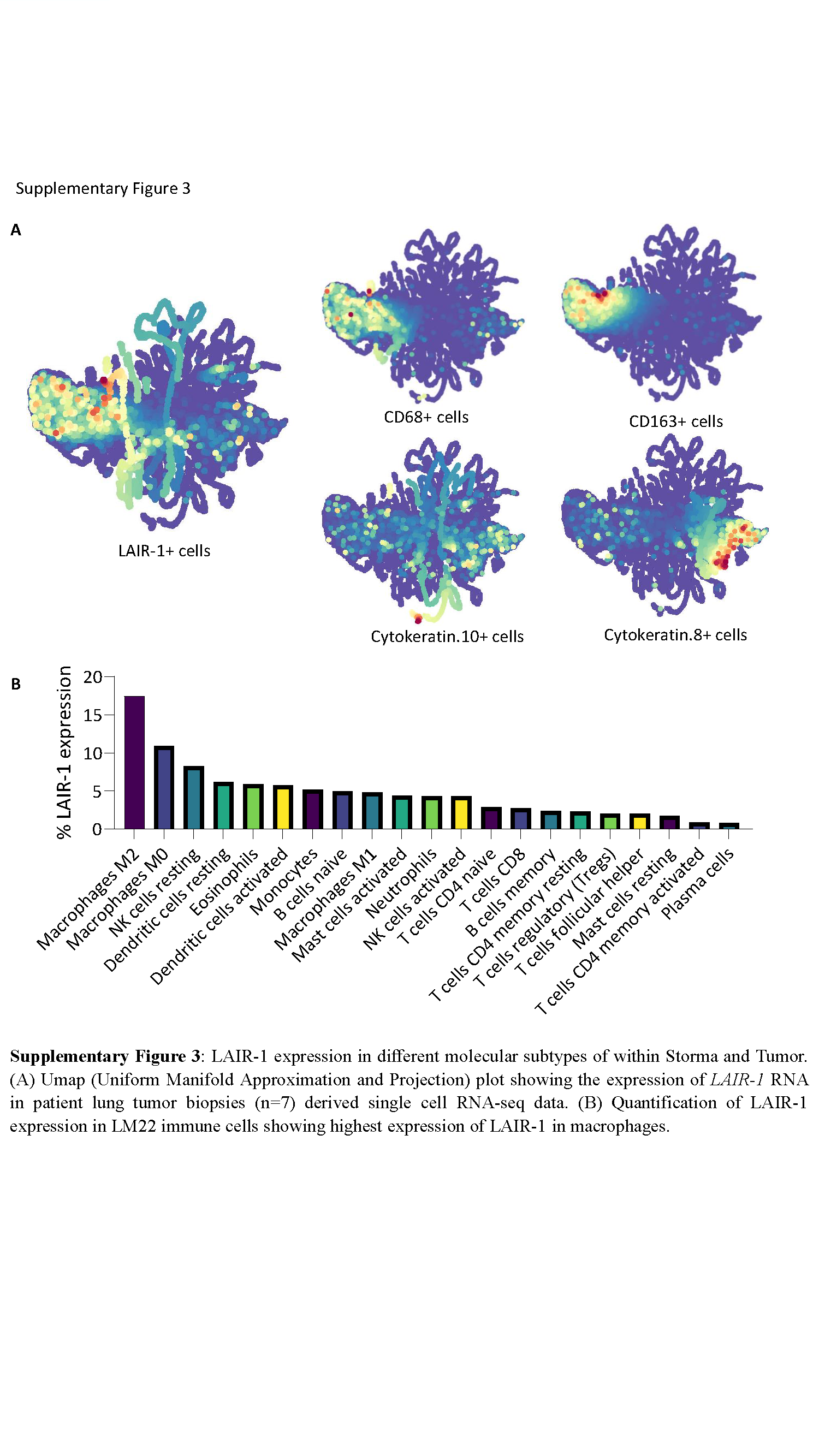

Supplement: Supplementary Figure FS3 — LAIR-1 expression in different molecular subtypes of within Storma and Tumor. (A) Umap (Uniform Manifold Approximation and Projection) plot showing the expression of LAIR-1 RNA in patient lung tumor biopsies (n=7) derived single cell RNA-seq data. (B) Quantification of LAIR-1 expression in LM22 immune cells showing highest expression of LAIR-1 in macrophages. [file crc-22-0334-s04.png]

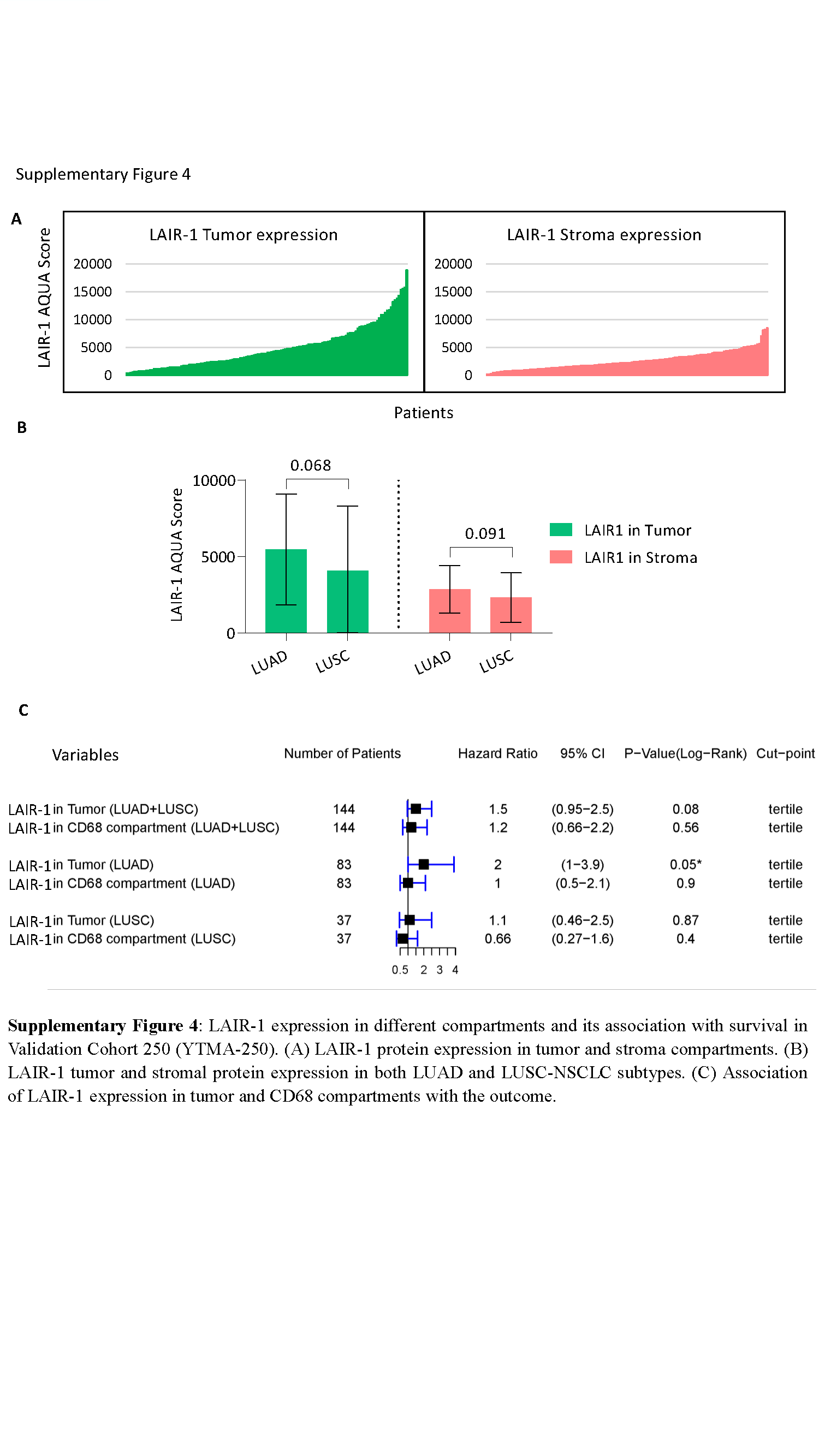

Supplement: Supplementary Figure FS4 — LAIR-1 expression in different compartments and its association with survival in Validation Cohort 250 (YTMA-250). (A) LAIR-1 protein expression in tumor and stroma compartments. (B) LAIR-1 tumor and stromal protein expression in both LUAD and LUSC-NSCLC subtypes. (C) Association of LAIR-1 expression in tumor and CD68 compartments with the outcome. [file crc-22-0334-s05.png]
