# Supplementary material for: Quantitative, Spatially Defined Expression of Leukocyte-associated Immunoglobulin-like Receptor in Non–small Cell Lung Cancer
Source: Cancer Res Commun. 2023 Mar 21;3(3):471–82. doi: 10.1158/2767-9764.CRC-22-0334 (PMC10029762; doi:10.1158/2767-9764.CRC-22-0334)
Supplement: Supplementary Table TS1 — Clinicopathological characteristics of NSCLC validation cohort (YTMA-250). [file crc-22-0334-s02.pptx]

## Slide 1
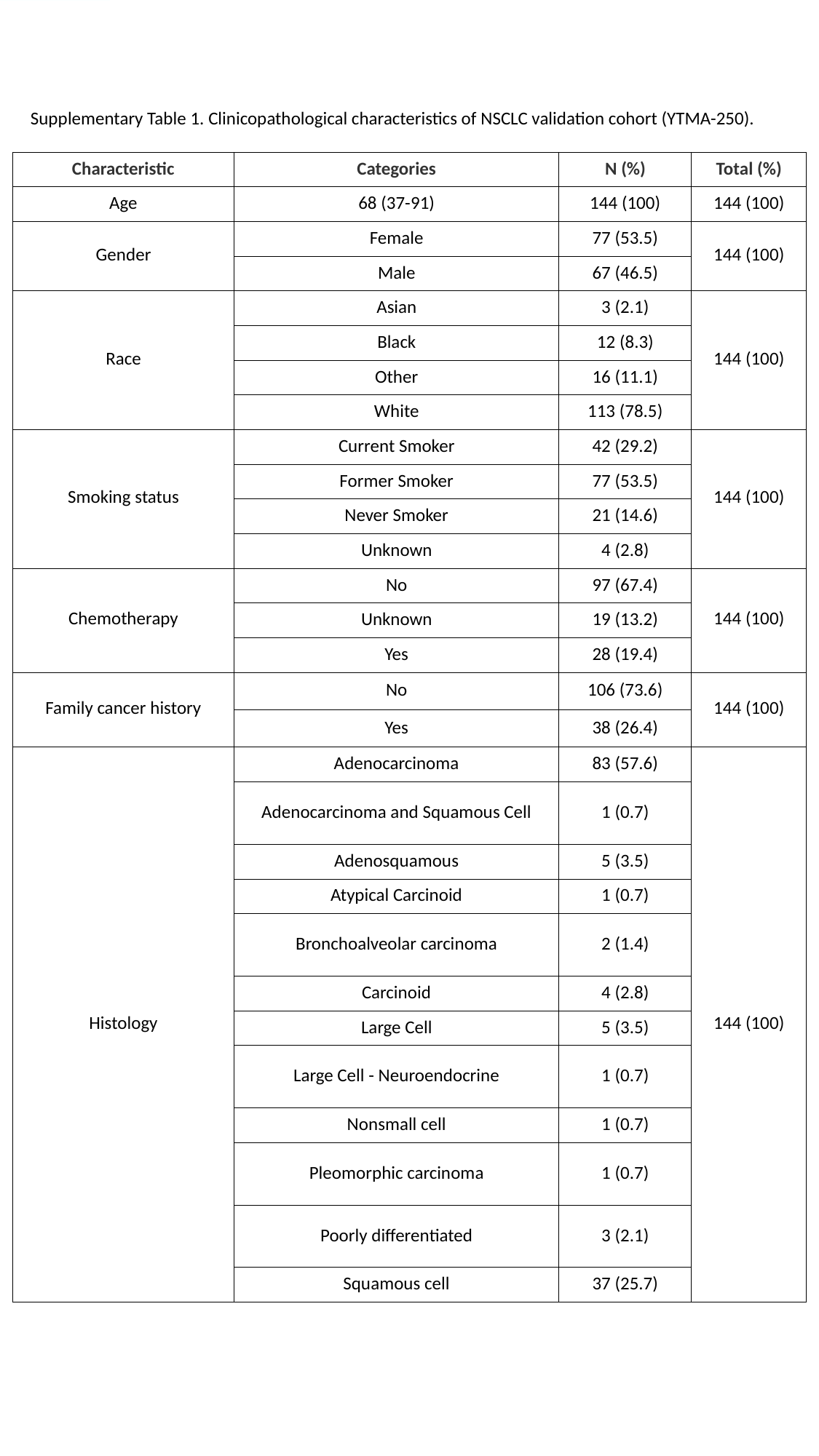

Supplementary Table 1. Clinicopathological characteristics of NSCLC validation cohort (YTMA-250).
| Characteristic | Categories | N (%) | Total (%) |
| --- | --- | --- | --- |
| Age | 68 (37-91) | 144 (100) | 144 (100) |
| Gender | Female | 77 (53.5) | 144 (100) |
| | Male | 67 (46.5) | |
| Race | Asian | 3 (2.1) | 144 (100) |
| | Black | 12 (8.3) | |
| | Other | 16 (11.1) | |
| | White | 113 (78.5) | |
| Smoking status | Current Smoker | 42 (29.2) | 144 (100) |
| | Former Smoker | 77 (53.5) | |
| | Never Smoker | 21 (14.6) | |
| | Unknown | 4 (2.8) | |
| Chemotherapy | No | 97 (67.4) | 144 (100) |
| | Unknown | 19 (13.2) | |
| | Yes | 28 (19.4) | |
| Family cancer history | No | 106 (73.6) | 144 (100) |
| | Yes | 38 (26.4) | |
| Histology | Adenocarcinoma | 83 (57.6) | 144 (100) |
| | Adenocarcinoma and Squamous Cell | 1 (0.7) | |
| | Adenosquamous | 5 (3.5) | |
| | Atypical Carcinoid | 1 (0.7) | |
| | Bronchoalveolar carcinoma | 2 (1.4) | |
| | Carcinoid | 4 (2.8) | |
| | Large Cell | 5 (3.5) | |
| | Large Cell - Neuroendocrine | 1 (0.7) | |
| | Nonsmall cell | 1 (0.7) | |
| | Pleomorphic carcinoma | 1 (0.7) | |
| | Poorly differentiated | 3 (2.1) | |
| | Squamous cell | 37 (25.7) | |
